# Supplementary material for: Chenodeoxycholic Acid-Amikacin Combination Enhances Eradication of Staphylococcus aureus
Source: Microbiol Spectr. 2023 Jan 10;11(1):e02430-22. doi: 10.1128/spectrum.02430-22 (PMC9927322; doi:10.1128/spectrum.02430-22)
Supplement: Supplemental file 1 — Supplemental material. Download spectrum.02430-22-s0001.pdf, PDF file, 2.2 MB [file spectrum.02430-22-s0001.pdf]

Supplementary Information for

Chenodeoxycholic acid and amikacin combination enhance eradication of *Staphylococcus aureus*

Kaiyu Cui[1†], Weifeng Yang[1†], Zhiyuan Liu[2], Guijian Liu[2], Dongying Li[1], Yanan Sun[1], Gaiying He[1], Shuhua Ma[1], Yu Cao[3], Xuefan Jiang[4], Sylvie Chevalier[5], Pierre Cornelis[5], Qing Wei[6*] and Yi Wang[1*]

*Corresponding author: Email: vubwqing@hotmail.com (Q.W.) and prof.wangyi@foxmail.com (Y.W.)
†These authors have contributed equally to this work.

This PDF file includes:

Supplementary Text
Figs. S1 to S7#
Tables S1 to S4#

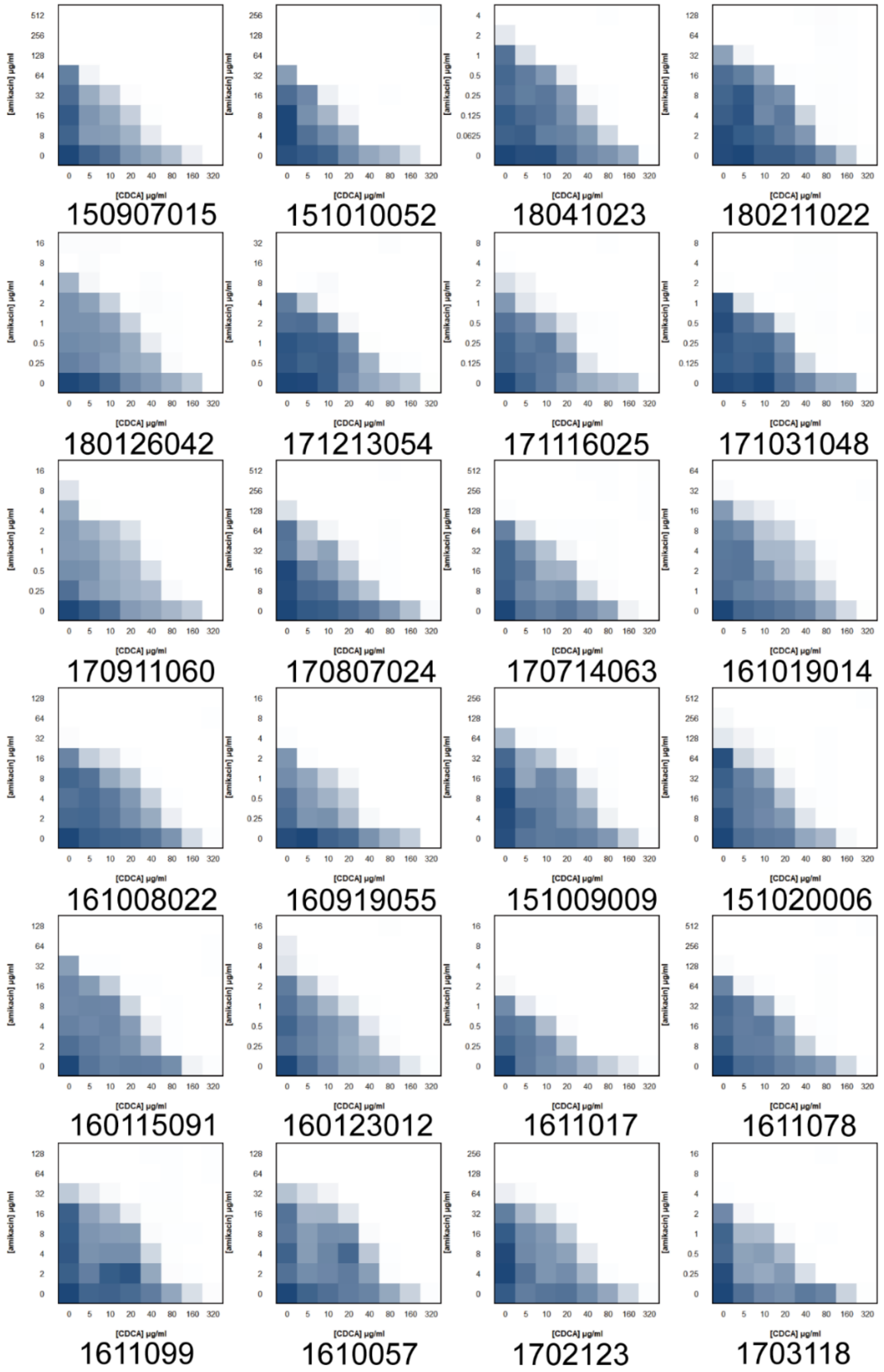

Fig. S1. CDCAA is effective against clinical MRSA strains.

CDCA growth inhibition checkerboards in combination with aminoglycosides treated 24 clinical MRSA strains.

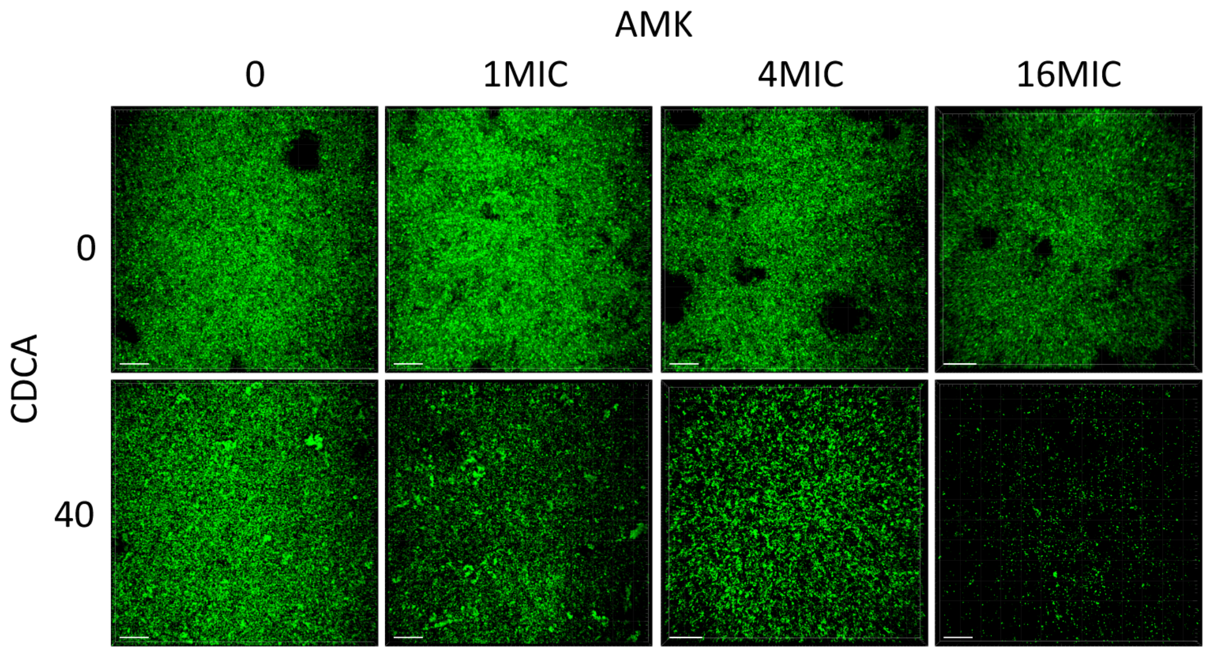

Fig. S2. CDCAA is effective against MRSA biofilms.
Representative separate green fluorescence (live bacteria) channels Z-axis (25 µm) overlay image of MRSA ATCC 43300 biofilms (triplicates) treated with amikacin alone (top) or in combination with CDCA (bottom). The scale bar is 70 µm. The fluorescence contrast values of each image was set at the same condition with a minimum of 500 and maximum of 2000 a.u. (arbitrary unit).

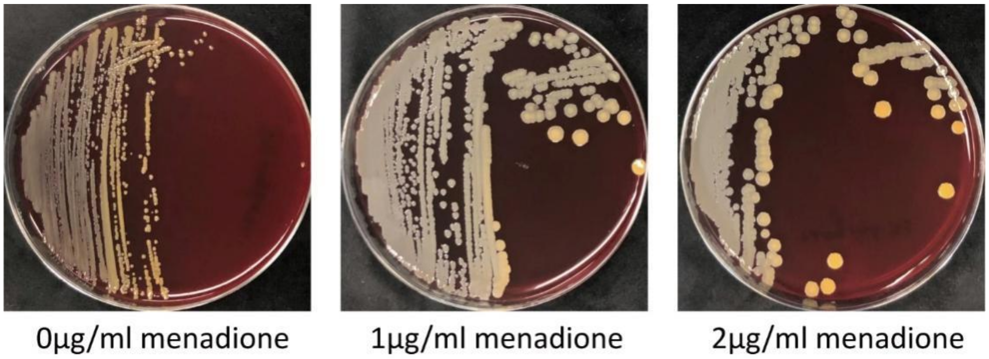

Fig. S3. Restoration of auxotroph of *S. aureus* SCVs.

Menadione (1 μg/ml and 2 μg/ml) was used to restore the auxotroph phenotype of *S. aureus* SCVs.

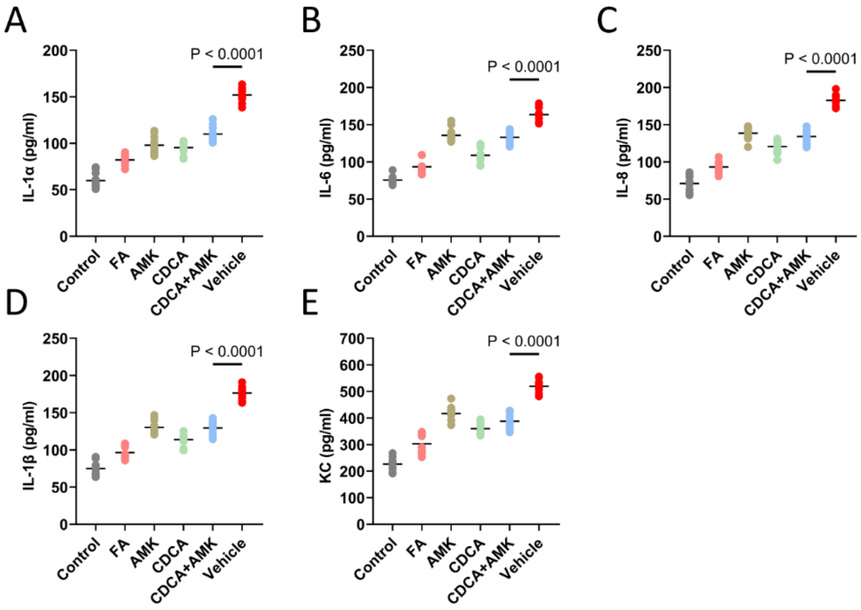

Fig. S4. ***In vivo*** **pro-inflammatory activity of health and injured skin.**

Cytokines levels were determined by ELISA. Data are shown as mean ± SEM ($n = 11$ mice per group). Significance was tested by one-way ANOVA in ranks with Kruskal-Wallis test ($p < 0.05$ between each group). IL-1α (A), IL-6 (B), IL-8 (C), IL-1β (D), and KC (E).

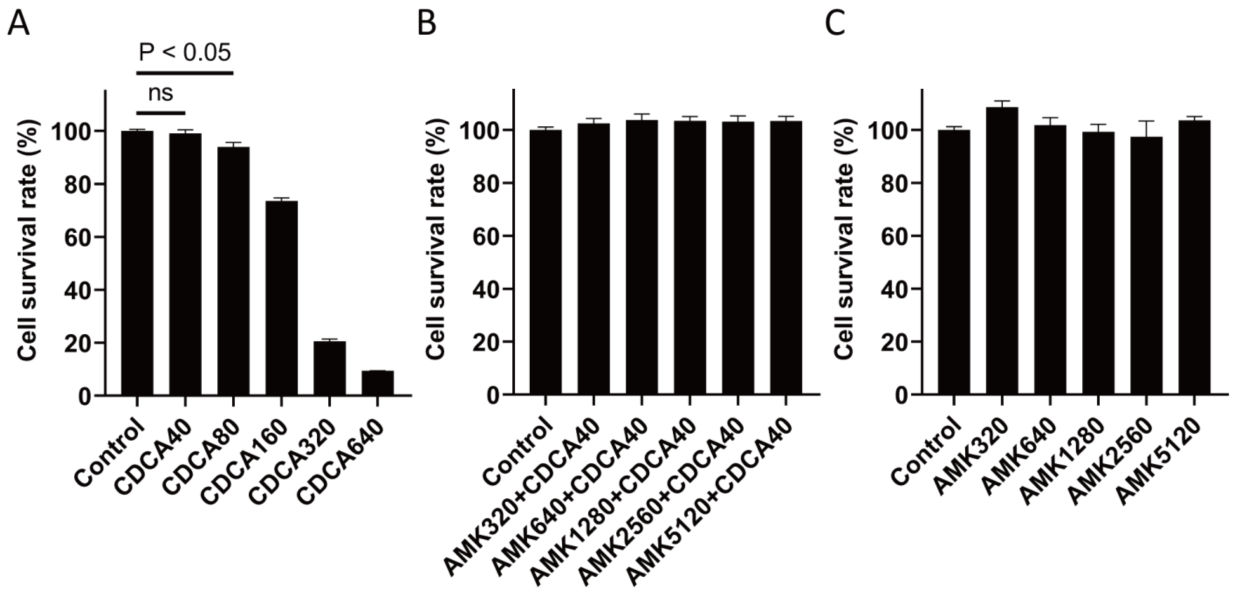

Fig. S5. CDCA does not inhibit the viability of mammalian cell.
CDCA alone (A) or CDCAA combination (B) or amikacin alone (C) at different concentrations showed no inhibitory effect on cell line HEI-OC1.

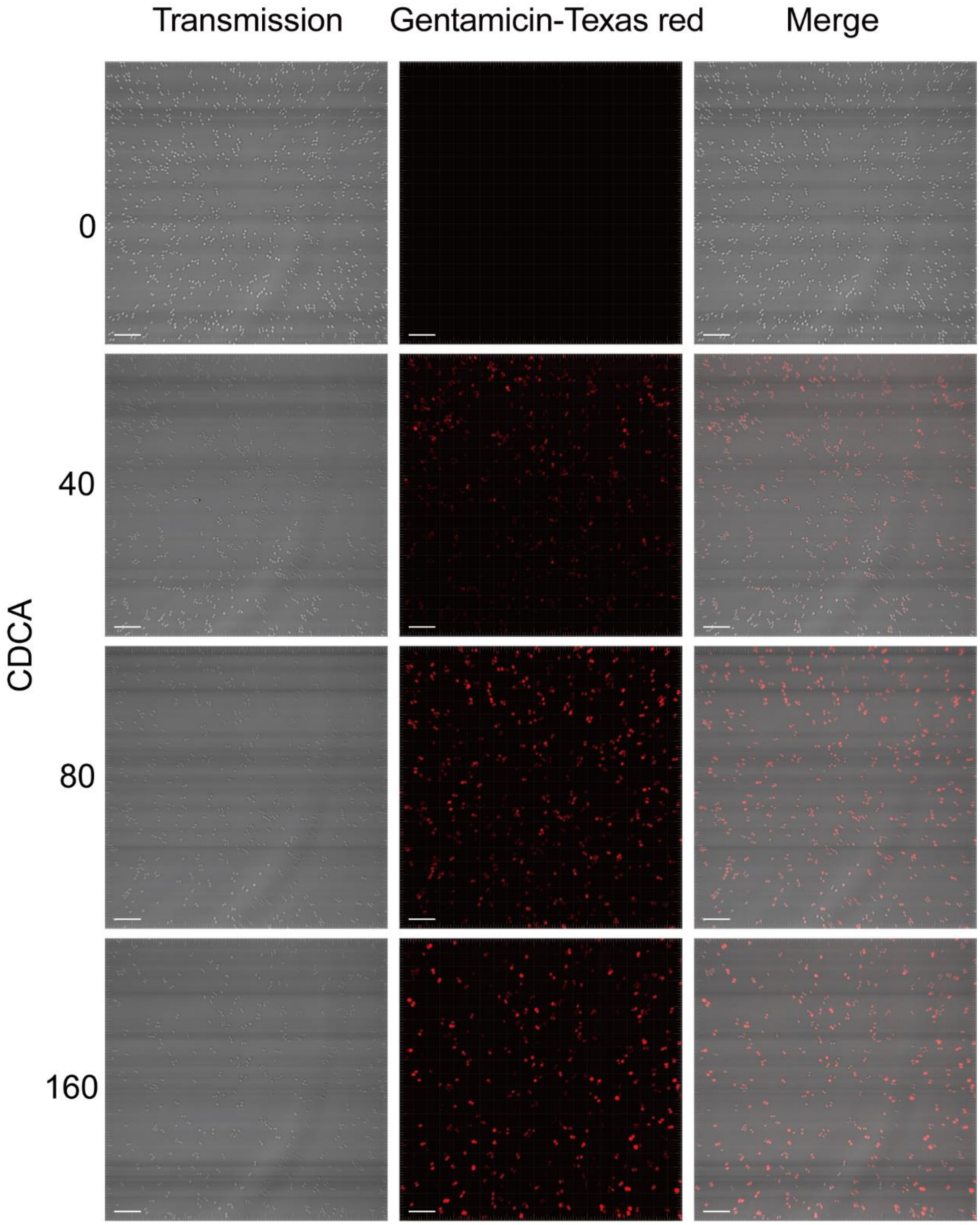

Fig. S6. CDCA increases gentamicin uptake.
Representative confocal fluorescence images of intracellular uptake of Texas red-gentamicin by MRSA ATCC 43300 with (0, 40, 80, and 160 μg/ml) or without CDCA treatment. The scale bar is 20 μm.

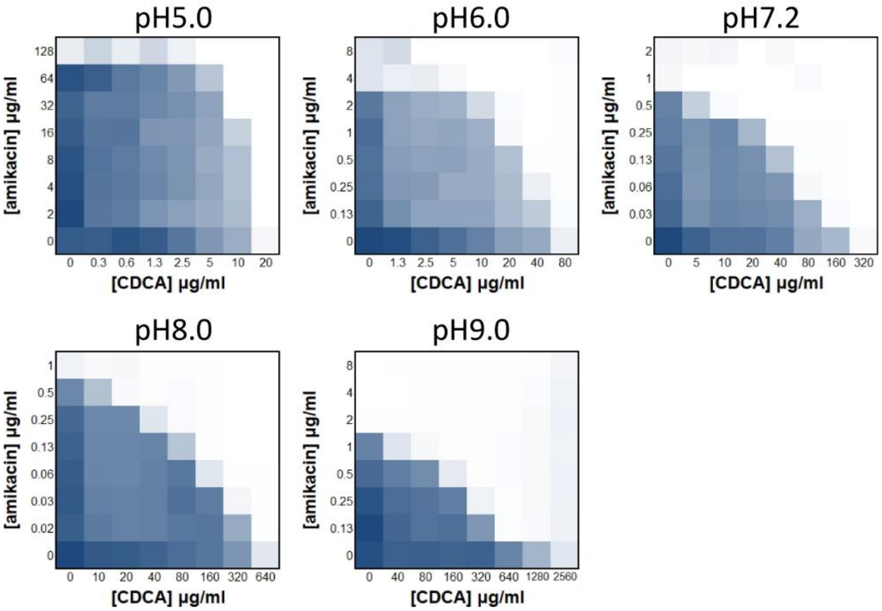

Fig. S7. The synergy between CDCA and amikacin at different pH conditions (pH 5.0~9.0).

Table S1. Minimal inhibitory concentration (MIC) of FA, CDCA and UDCA of 24 clinical MRSA strains.

	MIC (μg mL^{-1})		
**Clinical MRSA strain**	**FA**	**CDCA**	**UDCA**
150907015	0.5	320	1280
151010052	0.5	320	1280
180410023	0.25	320	1280
180211022	< 0.0625	320	1280
180126042	0.125	320	1280
171213054	0.5	320	1280
171116025	0.125	320	1280
171031048	0.25	320	1280
170911060	0.5	320	1280
170807024	1	320	1280
170714063	0.5	320	1280
161019014	**1024**	320	1280
161008022	**1024**	320	1280
160919055	0.5	320	1280
151009009	1	320	1280
151020006	0.5	320	1280
160115091	1	320	1280
160123012	0.5	320	1280
1611017	**512**	320	1280
1611078	**512**	320	1280
1611099	**512**	320	1280
1610057	0.125	320	1280
1702123	**1024**	320	1280
1703118	**512**	320	1280

Table S2. FICI of UDCA for aminoglycosides.

Antibiotics	*S. aureus* ATCC 25923	MRSA ATCC 43300
Amikacin	0.3125	0.3125
Etimycin	0.375	0.375
Gentamycin	0.375	0.375
Kanamycin	0.375	0.375
Tobramycin	0.375	0.375

Table S3. CDCA-treated *S. aureus* regulon in exponential growth phase.

Gene ID[a]	Name[a]	log2FC[b]	*P* value	Product[a]
SACOL0095	*spa*	-4.24	3.25E-11	immunoglobulin G binding protein A precursor
SACOL1421		-4.07	3.48E-11	phosphate ABC transporter, ATP-binding protein
SACOL1420		-3.71	3.83E-15	phosphate transport system protein PhoU, putative
SACOL1422		-3.44	1.53E-09	phosphate ABC transporter, permease protein
SACOL1424		-3.17	1.47E-09	phosphate ABC transporter, phosphate-binding protein
SACOL1423		-2.90	2.05E-06	phosphate ABC transporter, permease protein
SACOL2676	*sasA*	-2.59	2.83E-13	LPXTG cell wall surface anchor family protein
SACOL1173	*hlY*	-2.46	1.05E-07	alpha-hemolysin precursor
SACOL0205	*pflA*	-2.36	4.96E-05	pyruvate formate-lyase-activating enzyme
SACOL2547		-2.27	7.43E-06	hypothetical protein
SACOL0118	*sodA1*	-2.10	4.17E-10	superoxide dismutase
SACOL0204	*pflB*	-2.01	0.000402	formate acetyltransferase
SACOL1076	*purS*	-1.96	0.000241	phosphoribosylformylglycinamidine synthase, PurS protein
SACOL0248	*lrgB*	-1.79	0.002266	lrgB protein
SACOL2357		-1.78	4.84E-10	ABC transporter, permease protein
SACOL2635	*nrdD*	-1.76	7.08E-05	anaerobic ribonucleoside-triphosphate reductase
SACOL2671		-1.72	4.68E-10	secA family protein
SACOL1060		-1.69	8.35E-06	transcriptional regulator, MarR family
SACOL0608	*sdrC*	-1.57	6.92E-05	sdrC protein
SACOL1739		-1.56	2.03E-06	sensory box histidine kinase PhoR
SACOL0238		-1.56	2.07E-09	teichoic acid biosynthesis protein, putative
SACOL2605		-1.55	1.76E-08	conserved hypothetical protein
SACOL1077	*purQ*	-1.55	5.95E-05	phosphoribosylformylglycinamidine synthase I
SACOL2176	*opuD2*	-1.53	0.000548	osmoprotectant transporter, BCCT family
SACOL0609	*sdrD*	-1.52	1.49E-06	sdrD protein
SACOL2670		-1.50	2.42E-07	glycosyl transferase, group 1 family protein
SACOL0236		-1.47	9.06E-08	4-diphosphocytidyl-2C-methyl-D-erythritol synthase, putative
SACOL2634	*nrdG*	-1.46	0.003431	anaerobic ribonucleoside-triphosphate reductase activating protein
SACOL1740	*phoP*	-1.46	5.83E-05	alkaline phosphatase synthesis transcriptional regulatory protein PhoP
SACOL1074	*purK*	-1.45	0.000264	phosphoribosylaminoimidazole carboxylase, ATPase subunit
SACOL1078	*purL*	-1.42	1.29E-05	phosphoribosylformylglycinamidine synthase II
SACOL1075	*purC*	-1.41	0.000345	phosphoribosylaminoimidazole-succinocarboxamide synthase
SACOL2173		-1.41	5.40E-05	alkaline shock protein 23
SACOL2356		-1.32	0.000149	ABC transporter, ATP-binding protein
SACOL0237		-1.31	1.55E-07	alcohol dehydrogenase, zinc-containing
SACOL1933		-1.30	0.000151	ThiJ/PfpI family protein
SACOL0317		-1.29	0.000889	lipase precursor, interruption-N
SACOL2327	*hutG*	-1.28	0.000153	formiminoglutamase
SACOL0922		-1.27	5.20E-06	conserved hypothetical protein
SACOL2579		-1.26	4.87E-05	phytoene dehydrogenase
SACOL0921		-1.25	1.88E-05	CBS domain protein
SACOL2072		-1.24	1.70E-07	ATP-dependent RNA helicase, DEAD/DEAH box family

SACOL0200		-1.23	0.000556	phosphoglycerate transporter family protein
SACOL2175		-1.22	2.13E-05	conserved hypothetical protein
SACOL0119	*sasD*	-1.21	0.000826	cell wall surface anchor family protein
SACOL2669		-1.18	0.000163	conserved hypothetical protein
SACOL0271		-1.18	0.001623	conserved hypothetical protein
SACOL0336		-1.18	0.003254	conserved hypothetical protein
SACOL1090		-1.15	1.92E-06	conserved hypothetical protein
SACOL2622	*fdaB*	-1.14	0.00014	fructose-bisphosphate aldolase, class I
SACOL2578		-1.14	9.74E-05	glycosyl transferase, group 2 family protein
SACOL0677		-1.13	0.002603	conserved hypothetical protein
SACOL2174		-1.12	6.31E-08	conserved hypothetical protein
SACOL0764		-1.11	0.000654	glycosyl transferase, group 2 family protein
SACOL0698	*tagD*	-1.11	0.000102	glycerol-3-phosphate cytidylyltransferase
SACOL1828		-1.07	0.000451	conserved hypothetical protein
SACOL0976		-1.06	5.63E-05	hydrolase, haloacid dehalogenase-like family
SACOL0868		-1.05	5.27E-06	conserved hypothetical protein
SACOL0975		-1.03	0.000351	coenzyme A disulfide reductase
SACOL2361		-1.03	6.32E-05	hypothetical protein
SACOL1079	*purF*	-1.00	0.000245	amidophosphoribosyltransferase
SACOL1208	*lspA*	1.02	0.00043	signal peptidase II
SACOL2164		1.03	2.12E-05	conserved hypothetical protein
SACOL1455		1.04	5.91E-06	carboxyl-terminal protease
SACOL2449		1.05	0.003211	drug transporter, putative
SACOL1329	*femC*	1.06	6.62E-05	glutamine synthetase FemC
SACOL1943	*vraS*	1.06	9.92E-05	sensor histidine kinase VraS
SACOL1365		1.07	0.001855	hydrolase, haloacid dehalogenase-like family
SACOL2073	*murF*	1.08	0.001706	UDP-N-acetylmuramoyl-tripeptideD-alanyl-D-alanine ligase
SACOL1573		1.09	0.000411	
SACOL2721	*nixA*	1.10	0.000461	high-affinity nickel-transport protein
SACOL1320	*glpK*	1.12	0.000527	glycerol kinase
SACOL1786	*ccpA*	1.12	0.000406	catabolite control protein A
SACOL1066		1.12	0.001451	fmt protein
SACOL1225		1.12	6.56E-05	hypothetical protein
SACOL1732		1.13	0.00037	replication initiation and membrane attachment protein
SACOL0263	*lytM*	1.16	0.002143	peptidoglycan hydrolase
SACOL0320		1.18	2.95E-05	hypothetical protein
SACOL0630		1.20	0.003621	amino acid permease
SACOL1787		1.21	0.002566	chorismate mutase/phospho-2-dehydro-3-deoxyheptonate aldolase
SACOL1956		1.23	0.003211	conserved hypothetical protein
SACOL1733		1.23	0.000479	ATP cone domain protein
SACOL2436		1.23	3.31E-05	conserved hypothetical protein
SACOL1945		1.25	0.000735	conserved hypothetical protein
SACOL0884		1.25	4.56E-05	ABC transporter, substrate-binding protein
SACOL0151	*cap5P*	1.26	0.000259	UDP-N-acetylglucosamine 2-epimerase Cap5P
SACOL1673	*alaS*	1.28	0.00249	alanyl-tRNA synthetase

SACOL0147	*cap5L*	1.30	0.000382	capsular polysaccharide biosynthesis protein Cap5L
SACOL1362	*hom*	1.31	2.03E-06	homoserine dehydrogenase
SACOL0222	*ldh1*	1.31	0.003827	L-lactate dehydrogenase
SACOL1319	*glpF*	1.33	0.003567	glycerol uptake facilitator protein
SACOL0913		1.36	0.004651	conserved hypothetical protein
SACOL0148	*cap5M*	1.37	0.000279	capsular polysaccharide biosynthesis galactosyltransferase Cap5M
SACOL1363	*thrC*	1.38	0.000167	threonine synthase
SACOL0149	*cap5N*	1.40	9.07E-05	capsular polysaccharide biosynthesis protein Cap5N
SACOL0150	*cap5O*	1.40	0.000278	capsular polysaccharide biosynthesis protein Cap5O
SACOL1328	*glnR*	1.41	3.35E-11	glutamine synthetase repressor
SACOL2197		1.41	0.003202	surface protein, putative
SACOL2048	*leuC*	1.42	0.000661	3-isopropylmalate dehydratase, large subunit
SACOL0301		1.43	0.00132	formate/nitrite transporter family protein
SACOL1364	*thrB*	1.43	2.33E-05	homoserine kinase
SACOL2074		1.44	6.17E-05	D-alanineD-alanine ligase
SACOL1944		1.46	0.000145	conserved hypothetical protein
SACOL0452	*ahpC*	1.46	0.001038	alkyl hydroperoxide reductase, subunit C
SACOL1216	*pyrF*	1.50	0.005294	orotidine 5'-phosphate decarboxylase
SACOL2047	*leuB*	1.54	0.000489	3-isopropylmalate dehydrogenase
SACOL0166		1.58	1.11E-05	conserved hypothetical protein
SACOL1217	*pyrE*	1.59	0.002737	orotate phosphoribosyltransferase
SACOL0767		1.61	0.00135	conserved hypothetical protein
SACOL2046	*leuA*	1.63	0.000166	2-isopropylmalate synthase
SACOL1387		1.64	0.001532	conserved hypothetical protein
SACOL2531		1.64	0.000602	transcriptional regulator, MarR family
SACOL0502		1.67	0.000952	cysteine synthase/cystathionine beta-synthase family protein
SACOL0762		1.67	0.000442	hemolysin, putative
SACOL2382		1.68	6.22E-05	proton/sodium-glutamate symport protein
SACOL2584	*isaA*	1.71	1.62E-06	immunodominant antigen A
SACOL0451	*ahpF*	1.71	0.000377	alkyl hydroperoxide reductase, subunit F
SACOL1215	*carB*	1.72	0.000163	carbamoyl-phosphate synthase, large subunit
SACOL1705		1.74	1.76E-08	hypothetical protein
SACOL1213	*pyrC*	1.77	0.001631	dihydroorotase
SACOL2465		1.77	0.000173	addiction module antitoxin, Axe family
SACOL2464		1.80	4.76E-05	addiction module toxin, Txe/YoeB family
SACOL2138		1.80	0.000193	cation efflux family protein
SACOL2131		1.82	0.004701	Dps family protein
SACOL2557		1.86	0.000545	conserved domain protein
SACOL1212	*pyrB*	1.90	3.71E-05	aspartate carbamoyltransferase
SACOL2700	*hisB*	1.93	0.000891	imidazoleglycerol-phosphate dehydratase
SACOL2701		1.98	5.53E-05	histidinol-phosphate aminotransferase, putative
SACOL2704		2.05	3.20E-08	conserved hypothetical protein
SACOL2703	*hisG*	2.07	1.39E-06	ATP phosphoribosyltransferase
SACOL1214	*carA*	2.08	9.05E-06	carbamoyl-phosphate synthase, small subunit
SACOL2581		2.11	3.50E-09	staphyloxanthin biosynthesis protein

SACOL2571		2.17	0.00297	conserved hypothetical protein
SACOL1622	*glyS*	2.22	2.49E-05	glycyl-tRNA synthetase
SACOL2702	*hisD*	2.25	2.32E-06	histidinol dehydrogenase
SACOL0723		2.31	0.000439	LysM domain protein
SACOL2137	*czrA*	2.37	1.58E-07	transcriptional regulator CzrA
SACOL1383	*mscL*	2.45	9.35E-06	large conductance mechanosensitive channel protein
SACOL_SatmRNA1		2.47	1.61E-05	sRNA
SACOL1952		2.62	9.25E-12	ferritins family protein
SACOL2088		2.80	9.96E-07	sceD protein, putative
SACOL0009	*serS*	2.86	5.60E-08	seryl-tRNA synthetase
SACOL2529		3.05	2.48E-07	phospholipase/carboxylesterase family protein
SACOL2530		3.43	1.34E-11	conserved hypothetical protein
SACOL2295		3.46	8.05E-07	staphyloxanthin biosynthesis protein, putative
SACOL0625		3.73	2.86E-10	conserved hypothetical protein

a. Locus tag, gene name, gene product, and functional classification were extracted from AureoWiki (https://aureowiki.med.uni-greifswald.de/).
b. FC, fold change (log2 ratio).

Table S4. Strains used in this study.

Strain name	Features	References
S. aureus strains		
ATCC 43300	MRSA	ATCC
ATCC 25923	MSSA	Lab collection
SCV	Generated from ATCC 43300	This study
Persister	Generated from ATCC 43300	This study
150907015	Antibiotics resistance: penicillin-G, oxacillin, amikacin	Clinical laboratory of Guanganmen Hospital
151010052	Antibiotics resistance: penicillin-G, oxacillin, amikacin	Clinical laboratory of Guanganmen Hospital
180410023	Antibiotics resistance: penicillin-G, oxacillin	Clinical laboratory of Guanganmen Hospital
180211022	Antibiotics resistance: penicillin-G, oxacillin, amikacin	Clinical laboratory of Guanganmen Hospital
180126042	Antibiotics resistance: penicillin-G, oxacillin	Clinical laboratory of Guanganmen Hospital
171213054	Antibiotics resistance: penicillin-G, oxacillin	Clinical laboratory of Guanganmen Hospital
171116025	Antibiotics resistance: penicillin-G, oxacillin	Clinical laboratory of Guanganmen Hospital
171031048	Antibiotics resistance: penicillin-G, oxacillin	Clinical laboratory of Guanganmen Hospital
170911060	Antibiotics resistance: penicillin-G, oxacillin	Clinical laboratory of Guanganmen Hospital
170807024	Antibiotics resistance: penicillin-G, oxacillin, amikacin	Clinical laboratory of Guanganmen Hospital
170714063	Antibiotics resistance: penicillin-G, oxacillin, amikacin	Clinical laboratory of Guanganmen Hospital
161019014	Antibiotics resistance: penicillin-G, oxacillin; Intermediate: amikacin	Clinical laboratory of Guanganmen Hospital
161008022	Antibiotics resistance: penicillin-G, oxacillin; Intermediate: amikacin	Clinical laboratory of Guanganmen Hospital
160919055	Antibiotics resistance: penicillin-G, oxacillin	Clinical laboratory of Guanganmen Hospital
151009009	Antibiotics resistance: penicillin-G, oxacillin, amikacin	Clinical laboratory of Guanganmen Hospital
151020006	Antibiotics resistance: penicillin-G, oxacillin, amikacin	Clinical laboratory of Guanganmen Hospital
160115091	Antibiotics resistance: penicillin-G, oxacillin, amikacin	Clinical laboratory of Guanganmen Hospital
160123012	Antibiotics resistance: penicillin-G, oxacillin	Clinical laboratory of Guanganmen Hospital
1611017	Antibiotics resistance: penicillin-G, oxacillin, amikacin	Clinical laboratory of Dongzhimen Hospital
1611078	Antibiotics resistance: penicillin-G, oxacillin	Clinical laboratory of Dongzhimen Hospital
1611099	Antibiotics resistance: penicillin-G, oxacillin, amikacin	Clinical laboratory of Dongzhimen Hospital
1610057	Antibiotics resistance: penicillin-G, oxacillin, amikacin	Clinical laboratory of Dongzhimen Hospital
1702123	Antibiotics resistance: penicillin-G, oxacillin, amikacin	Clinical laboratory of Dongzhimen Hospital
1703118	Antibiotics resistance: penicillin-G, oxacillin	Clinical laboratory of Dongzhimen Hospital
